# Corrélation entre l’imagerie par résonnance magnétique, l’extemporanée et l’histologie définitive des tumeurs parotidiennes: série de cas

**DOI:** 10.11604/pamj.2020.37.80.21192

**Published:** 2020-09-22

**Authors:** Najib Zouhair, Sanaa Mallouk, Youssef Oukessou, Sami Rouadi, Redallah Larbi Abada, Mohamed Roubal, Mohamed Mahtar

**Affiliations:** 1 Service d´Otorhinolaryngologie et Chirurgie Cervico-Faciale, Hôpital 20 Août, Centre Hospitalier Universitaire Ibn Rochd, Université Hassan II, Casablanca, Maroc

**Keywords:** Extemporané, corrélation, sensibilité, tumeur parotidienne, Extemporaneous, correlation, susceptibility, parotid gland tumor

## Abstract

La pathologie tumorale de la glande parotide est complexe et pose des problèmes aussi bien diagnostiques que thérapeutiques. Notre objectif était d´évaluer la place de l'examen extemporané dans la prise en charge des tumeurs parotidiennes. Nous rapportons une étude analytique pro et rétrospective sur une série de cas présentant une tumeur parotidienne, colligés au service d'ORL et de chirurgie cervico-faciale du CHU de Casablanca, entre janvier 2012 et décembre 2015. Nous avons noté 72 cas de tumeurs parotidiennes. Le sexe-ratio (H/F) est de 0,94, il était de 0,76 pour les tumeurs bénignes et de 1,62 pour les tumeurs malignes. La moyenne d'âge de nos patients est de 47 ans (15-75 ans). Le délai moyen de consultation est de 40 mois. La symptomatologie clinique était dominée par la tuméfaction parotidienne (100%), la douleur chez 25% des patients, la paralysie faciale chez 6%, et les adénopathies cervicales chez 10%. L'échographie était demandée dans 80%. L'IRM était réalisée dans 26% des cas. Tous nos patients ont bénéficié d'un traitement chirurgical, 76% de parotidectomies exo-faciales et 24% de parotidectomies totales conservatrices. Ce traitement a été associé à un évidemment ganglionnaire dans 24% des cas et d´une radiothérapie dans 24%. L´examen extemporané a été réalisé chez 71% des patients, sa sensibilité était de 89% et sa spécificité était de 88%. Le diagnostic définitif de la nature histologique a été confirmé par l'examen anatomopathologique dans tous les cas. Nous avons confirmé la nature bénigne et maligne de la tumeur respectivement dans 71% et 29%. L'adénome pléomorphe a dominé les tumeurs bénignes (59%), alors que les lésions malignes étaient dominées par le carcinome muco-épidermoïde (38%). Les suites opératoires étaient marquées par: un discret hématome dans 4%, une paralysie faciale transitoire dans 15%, une surinfection de la plaie dans 3% et un syndrome de Frey post parotidectomie chez 3% des patients. Un cas de récidive labiale d´un carcinome à cellule acineuse. Aucun cas de décès n´a été noté. Les tumeurs de la glande parotide se caractérisent par une grande diversité histologique. La différenciation entre une tumeur maligne et une tumeur bénigne est souvent délicate. Actuellement l´IRM permet d´apporter plus de renseignements. L´extemporané est d´une aide précieuse en peropératoire qui nécessite une communication avec l´anatomopathologiste. Sa prise en charge est pluridisciplinaire, ORL, onco-radiothérapeute, anatomopathologiste et radiologue. Le pronostic dépend du type histologique, du stade évolutif et du traitement.

## Abstract (en)

Parotid gland tumor is complex and poses diagnostic and therapeutic problems. The purpose of this study was to assess the role of extemporaneous examination in the management of patients with parotid gland tumors. We report a pro and retrospective analytical study of a series of cases of salivary gland tumors, whose data were collected in the ENT and in the department of cervical-facial Surgery at the University Hospital in Casablanca, between January 2012 and December 2015. Seventy two cases of parotid tumors were recorded. The sex-ratio (H/F) was 0.94, 0.76 for patients with benign tumors and 1.62 for patients with malignant tumors. The average age was 47 years (15- 75 years). The median of consultation time was 40 months. Clinical symptoms were dominated by parotid swelling (100%), pain in 25% of patients, facial palsy in 6%, and cervical adenopathies in 10%. Ultrasound was recommended in 80% of patients. MRI was performed in 26% of cases. All patients underwent surgery, 76% of patients underwent exofacial parotidectomies and 24% total conservative parotidectomies. This treatment was associated with ganglion resection in 24% of cases and radiotherapy in 24% of cases. Extemporaneous examination was performed in 71% of patients, its susceptibility was 89% and its specificity 88%. Definitive histological diagnosis was confirmed by anatomopathological examination in all cases. We confirmed benign and malignant tumors in 71% and 29% of cases respectively. Benign tumors were dominated by pleomorphic adenoma (59%), while malignant lesions were dominated by mucoepidermoid carcinoma (38%). The postoperative course was marked by: discrete haematoma in 4% of cases, transient facial palsy in 15%, superinfection of the wound in 3% and post-parotidectomy Frey´s syndrome in 3% of patients. One patient had labial recurrence of acinar cell carcinoma. No cases of death were noted. Parotid gland tumors are characterized by a great histological variability. Differentiation between malignant tumor and benign tumor is often difficult. Currently, MRI is the imaging test of choice. Extemporaneous examinationis is very useful intraoperatively when it is necessary to communicate with the pathologist. Multidisciplinary approach is adopted including ENT, oncological, radiotherapeutic, pathological and radiological approaches. Prognosis depends on the histological type, the stage of progression and treatment.

## Introduction

Les tumeurs de la glandes parotide représentent les tumeurs des glandes salivaires les plus fréquentes néanmoins ces dernières demeure relativement rare représentant 3 à 4% de l'ensemble des tumeurs de la tête et du cou [1, 2]. Ces tumeurs sont caractérisées par une grande diversité histologique, mais ce sont les formes bénignes qui prédominent avec en chef de file l'adénome pléomorphe ou tumeur mixte [1-3]. La connaissance préopératoire de la nature de la tumeur guide le chirurgien dans sa décision chirurgicale; par conséquent, l´imagerie est devenue un véritable outil diagnostique pour les chirurgiens, l´échographie couplée ou non à la cytoponction, la TDM et l´IRM, sont les méthodes les plus fréquemment utilisées. Cependant, l´efficacité de chacune dans l´évaluation de la nature de la tumeur n´est pas encore bien définie. En effet, dans de nombreuses situations, où le chirurgien suspecte la malignité, les radiologues ne peuvent pas trancher entre tumeur maligne et bénigne; d´autre part, il existe parfois des discordances entre la réponse des radiologues et le diagnostic histopathologique, ce qui a motivé la réalisation de ce travail dont l´objectif est de déterminer la sensibilité et la spécificité de chaque examen radiologique et la corrélation entre le résultat de l´extemporané et de l´histologie définitive.

## Méthodes

Il s´agit d´une étude évaluative d´un test diagnostic pro et rétrospective portant sur 72 cas, menée au sein du service d'ORL et de chirurgie cervico-faciale du CHU de Casablanca, sur une période de quatre ans entre janvier 2012 et décembre 2015. Ont été inclus tous les patients admis au service ayant été suivis et traités pour une tumeur parotidienne. Le recueil des données a été réalisé à partir des registres d´admission, des dossiers d´hospitalisation des patients, des comptes rendus opératoires et des comptes rendus d´anatomie pathologique et de l´examen extemporané. Les variables que nous avons analysées étaient principalement l´âge, le sexe, les antécédents, les signes cliniques, les signes paracliniques, la prise en charge thérapeutique et la corrélation entre l´examen extemporané et l´histologie définitive. L´analyse statistique et le calcul des moyennes et des pourcentages sont obtenus à l´aide du Logiciel SPSS 2.0, ce qui nous a permis d'obtenir les résultats présentés dans le chapitre suivant.

## Résultats

Au cours d´une période de quatre ans, allant de janvier 2012 à décembre 2015, nous avions colligé 72 cas de tumeurs de la parotide. La moyenne d´âge de nos patients a été de 47 ans avec des extrêmes entre 15 et 75 ans. La tranche d´âge la plus touchée était entre 41 et 50 ans. Le sexe-ratio (H/F) est de 0,94(49% hommes et 51% femmes)

**Données cliniques:** le délai de consultation chez nos patients était de 44 mois pour les tumeurs bénignes et de 26 mois pour les tumeurs malignes. La tuméfaction de la région parotidienne a été un signe révélateur constant. Elle a été retrouvée chez 100% des patients. L'examen clinique de la région parotidienne avait montré une tuméfaction parotidienne chez la totalité des patients, cette tuméfaction était unilatérale pour tous nos patients, sa taille a varié entre 1 à 10 cm avec une moyenne de 3,4cm. La peau en regard de la tuméfaction parotidienne a été infiltrée chez 3% des patients. 65% des patients ont une tuméfaction du côté droit et 35% des patients ont une tuméfaction du côté gauche. Aucune atteinte parotidienne controlatérale ni des autres glandes salivaires n´a été notée. La consistance de la masse a été ferme chez 83% des patients, dure chez 8,5% des patients et molle chez 8,5% patients. La masse parotidienne était mobile par rapport aux deux plans chez 82% des patients, et fixé aux deux plans chez 18% des patients, dont 3% présentaient une infiltration de la peau. L´examen des aires ganglionnaires avait trouvé des adénopathies cervicales jugulocarotidiennes supérieures homolatérales chez 10% des patients. L'examen du conduit auditif externe et du cuir chevelu était normal chez tous nos patients.

### Données paracliniques

**Échographie cervicale:** parmi nos 72 malades, 80% des patients ont bénéficié de cet examen; elle avait montré la taille, les limites, et l'échostructure tumorale, celle-ci a été hypoéchogène chez 86% des patients, hétérogène et mal limitée chez 12% des patients, et kystique et mal limitée chez 2% des patients. Les adénopathies cervicales ont été visualisées dans 7% des cas. Sa sensibilité était de 85% pour les tumeurs bénignes.

**Tomodensitométrie cervico-faciale (TDM):** le scanner de la région parotidienne et de la région cervicale en coupes axiales et coronales sans et avec injection de produit de contraste est réalisé chez 66% des patients. Sa sensibilité était de 36% pour les tumeurs malignes. Il a évoqué une tumeur maligne chez 17% des patients devant l´aspect hétérogène de la tumeur, les limites irrégulières et un rehaussement massif à l´injection du produit de contraste, l´envahissement des structures adjacentes.

**Imagerie par résonnance magnétique (IRM):** l'IRM a été réalisée chez 26% de nos patients, elle avait pour objectif surtout d'analyser le signal de la tumeur parotidienne et ses rapports avec les structures de voisinage. L´IRM a évoqué une tumeur bénigne chez 63% des patients devant des formations nodulaires bien limitées et rehaussées de façon hétérogène à l´injection du Gadolinium. La nature maligne a été évoquée chez 37% des patients devant: un processus tissulaire hétérogène, mal limité, en hyposignal T2, chez 21% des patients. Une formation hétérogène à centre nécrosé avec adénopathies sous digastriques dans 16% des cas. Le diagnostic était concordant avec les résultats anatomopathologiques dans 100% des cas.

**Bilan d´extension:** en cas de cancer confirmé, un bilan d'extension est toujours réalisé pour rechercher une deuxième localisation. Le bilan d'extension a été clinique et para clinique, le scanner de la région parotidienne a fait l'essentiel du bilan d'extension locorégionale. Le bilan d'extension général a fait appel à l´examen clinique et à deux examens paracliniques: la radiographie pulmonaire, l'échographie abdominale.

**Classification TNM:** au terme du bilan d´extension, les patients présentant une tumeur maligne de la parotide-ont été classés selon la classification de l´UICC (Union Internationale Contre le Cancer) de 2002. La tumeur a été classée T3 chez 67% des patients, T2 chez 24% des patients et T4 chez 9% des patients. Le stade ganglionnaire été classé N0 chez 57% des patients, N1 chez 33% des patients et N2 chez 10% des patients. Aucun patient n´a présenté des métastases à distances au moment du diagnostic.

### Prise en charge thérapeutique

#### La chirurgie

##### Chirurgie sur la tumeur

**L´abord chirurgical:** nous avons réalisé 72 abords chirurgicaux de la région parotidienne. La voie d´abord utilisée chez tous nos patients était une voie de Redon.

**L´examen extemporané:** l´examen extemporané a été réalisé chez 71% des patients, il avait répondu bénin dans 61% des cas et malin dans 39% des cas.

**Chirurgie des aires ganglionnaires:** le geste chirurgical local était accompagné d´un curage ganglionnaire fonctionnel dans 24% des cas.

**Radiothérapie:** 24% des patients ont bénéficié d´une radiothérapie postopératoire. Le délai moyen entre la chirurgie et la radiothérapie était de 8 semaines.

**Chimiothérapie:** un cas de LMNH a bénéficié d´une chimiothérapie. Il a reçu le protocole: CHOP (Cyclophosphamide, Adriblastine, Oncovin, Prédnisone).

**Données anatomopathologiques:** l'analyse anatomopathologique des lésions a révélé une grande diversité histopathologique (Tableau 1, Tableau 2, Tableau 3), pour cela, nous avons adopté la classification histologique de l'OMS de 2005.

**Tableau 1:** répartition histologique des tumeurs épithéliales bénignes

Type histologique	Nombre	Pourcentage
Adénome pléomorphe	30	42%
Tumeur Warthin	9	13%
Cystadénome	6	8%
Adénome à cellule basale	2	3%
Myoépithéliome	1	1%
Oncocytome	1	1%
Total	**49**	**68%**

**Tableau 2:** répartition histologique des tumeurs épithéliales malignes

Type histologique	Nombre	Pourcentage
Lipome	2	3%
Lymphome	1	1%
Total	**3**	**4%**

**Tableau 3:** répartition histologique des tumeurs non épithéliales

Type histologique	Nombre	Pourcentage
Carcinome mucoépidermoïde	8	11%
Adénocarcinome	5	7%
Carcinome adénoïde kystique	3	4%
Carcinome épidermoïde	2	3%
Carcinome à cellules acineuses	2	3%
Total	20	28%

**Corrélation examen extemporané / histologie définitive:** parmi nos 72 patients, 71% ont bénéficié d´un examen extemporané. Il avait répondu “bénin” dans 61% des cas et “malin” dans 39% des cas. L´histologie définitive a trouvé 65% de tumeurs bénignes et 35% de tumeurs malignes. La comparaison entre les résultats de l´examen extemporané et celles de l´histologie définitive trouve, 31% de vrais positif (les tumeurs identifiées correctement comme malignes), 57% de vrais négatif (les tumeurs identifiées correctement comme bénignes), 8% de faux positif (les tumeurs identifiées faussement comme malignes) et 4% de faux négatif (les tumeurs identifiées faussement comme bénignes). Nous avons calculé la sensibilité (probabilité que la réponse soit « malin » si la tumeur est maligne) et la spécificité (probabilité que la réponse soit « bénin » si la tumeur est bénigne) de l´examen extemporané: la sensibilité était de 89% et la spécificité était de 88%. La valeur prédictive positive (probabilité que la tumeur soit maligne si la réponse est « malin ») était de 80%, la valeur prédictive négative (probabilité que la tumeur soit bénigne si la réponse est « bénin ») était de 94%.

### Données évolutives

**Évolution à court terme:** l'évolution postopératoire était bonne chez 75% des patients. Nous avons constaté 15% de cas de paralysie faciale transitoire, ayant régressé sous traitement médical et kinésithérapie. Aucun cas de paralysie faciale définitive n´a été noté. 3% des patients ont présenté une infection de la plaie, ils ont bénéficié de soins locaux quotidiens avec administration d'une antibiothérapie par voie orale, l'évolution était bonne. Un syndrome de Frey post parotidectomie était observé chez 3% des patients. 4% des patients ont présenté un hématome qui a été drainé chirurgicalement.

**Évolution à long terme:** pendant la durée de notre étude, 30 patients ont été perdus de vue, 32 patients ont été bien suivis. Un cas de récidive labiale d´un carcinome à cellule acineuse. Aucun cas de décès n´a été noté.

**Traitement des complications:** les patients ayant présenté une paralysie faciale post-opératoire ont bénéficié d´une corticothérapie per os et des soins oculaires associés à une kinésithérapie faciale.

## Discussion

Les tumeurs de la parotide sont rares, avec une incidence de 1 par 100.000 habitants par ans. Elles sont bénignes dans 80% des cas, et la parotide constitue leur siège de prédilection [4, 5]. Dans notre série la fréquence des tumeurs parotidiennes a été estimée à 18 nouveaux cas par an. L'âge moyen de découverte des tumeurs parotidiennes est de 45 ans, et le pic de fréquence se situe entre 5^e^et la 6^e^décade [4]. La sex-ratio est autour de 1 selon les auteurs, dans notre étude il est à 0,94.

### Examen clinique

**Douleur**: la douleur, peu fréquente, est notée, dans seulement 6% à 15% des cas de tumeurs malignes de la parotide selon les séries (Spiro [6], Eneroth [7]). La douleur est plus fréquente en cas de tumeur maligne mais elle n'est pas spécifique des carcinomes adénoïdes kystiques [8]. Elle peut être notée dans le cadre des tumeurs bénignes dans 5% [9]. Elle est considérée comme un facteur de mauvais pronostic et elle indique une atteinte nerveuse [10, 11]. Dans notre série 25% des patients avaient une douleur de la région parotidienne dont la plupart étaient des tumeurs malignes (67% de tumeurs maligne contre 33% de tumeurs bénignes).

**Paralysie faciale:** l'atteinte du nerf faciale est rapportée dans 12% à 22% des cas selon les séries (Pederson [12], Spiro [6]). Elle peut être en rapport avec une compression, une inflammation ou l'infiltration péri nerveuse [13]. Dans notre série la paralysie faciale a été retrouvée chez 6% des patients.

**La fixité de la tumeur par rapport aux plans de voisinage:** une tumeur fixée ou à l'origine d'une infiltration dermique est toujours fortement suspecte de malignité [8, 9, 14]. Elle est notée dans 58% des tumeurs selon Pederson [12]. Certaines tumeurs bénignes peuvent cependant s'enclaver dans l'espace mastoïdomandibulaire, sans que cette fixité ait une signification pronostique péjorative [8, 11]. Dans notre série la masse parotidienne était mobile par rapport aux deux plans chez 82% des patients, et fixé aux deux plans chez 18% des patients.

**Les adénopathies:** dans une série de Coiffier [15], les adénopathies ont été palpées chez 5 patients dont 3 se sont révélées être des métastases ganglionnaires. Przewozny [16] dans son étude, a noté l'existence d´adénopathie dans 23% des cas. Dans notre série l´examen des aires ganglionnaires avait trouvé des adénopathies cervicales jugulocarotidiennes supérieures homolatérales chez 10% des patients.

**Tuméfaction:** pour ce qui est des modes de révélation de la maladie, ils ont été dominés par la tuméfaction parotidienne retrouvée chez tous nos patients, soit un taux de l'ordre de 100%. Ce résultat est en accord avec celui décrit dans la série américaine, tunisienne et sénégalaise [14].

### Exploration paraclinique

**L'échographie:** longtemps considérée comme un examen clé de l'exploration des tumeurs des glandes salivaires [8], l'échographie est maintenant considérée comme un examen d'intérêt limité [17, 18]. La sensibilité de cet examen dans la détection des tumeurs du lobe superficiel de la parotide est proche de 100%, tout en différenciant ces lésions des lésions extra-glandulaires superficielles [8]. À l'inverse, l'examen est limité dans l'exploration du lobe profond à cause de la branche montante de la mandibule [8, 18, 19]. Dans notre série, 80% des patients ont bénéficié d'une échographie de la région parotidienne. Sa sensibilité était de 85% pour les tumeurs bénignes.

**La tomodensitométrie TDM:** la tomodensitométrie permet une évaluation précise du volume tumoral mais sa valeur diagnostique de nature tumorale apparait très inconstante [8, 18, 20]. Dans notre série: la TDM a été réalisé chez 66% des patients. Sa sensibilité était de 36% pour les tumeurs malignes. Le scanner en cas de diagnostic clinique reste le moyen idéal pour faire un bilan d´extension aux structures voisines, et estimer l´état des ganglions [21].

**L'imagerie par résonnance magnétique IRM:** l'IRM constitue l'examen de choix dans l'exploration des processus tumoraux parotidiens [8, 18]. Dans notre série, 26% des patients ont bénéficié de cet examen. Il a évoqué une tumeur bénigne chez 63% des patients et une tumeur maligne chez 37% des patients. La valeur prédictive du type histologique tumorale de l'IRM pour les tumeurs bénignes apparait assez fiable comprises entre 80 et 100% (particulièrement pour les adénomes pléomorphes), alors qu'elle est nulle pour le diagnostic histologique des tumeurs malignes [18].

**Caractéristiques de la lésion:** les critères morphologiques en faveur de malignité sont [18]: hyposignal en Tl et T2; signal très hétérogène; contours irréguliers; Infiltration des espaces adjacents; La multiplicité des lésions et la présence des adénopathies nécrotiques. Selon takashima [17] le critère le plus performant prédictif de malignité est l'hypo signal en T2 et l'hypo signal en T1 (sensibilité de 79%, spécificité de 82%, exactitude de 82%).

**Traitement:** dans notre série, la parotidectomie exo-faciale a été réalisé chez 76% des patients. L´examen extemporané a été réalisé chez 71% des patients, il avait répondu “bénin” dans 61% des cas et “malin” dans 39% des cas. Un curage ganglionnaire fonctionnel a été réalisé dans 24% des cas. Plusieurs auteurs [22, 23] ont pu observer un taux d'échec plus important chez les patients traités par chirurgie seule. Les patients traités par chirurgie seule présentent un taux d'échec local de 37% contre 23% pour ceux ayant eu une radiothérapie post-opératoire [24]. Dans notre série 24% des patients ont bénéficié d´une radiothérapie postopératoire.

**Corrélation examen extemporané / histologie définitive**: l´examen extemporané est un outil essentiel dans la prise en charge chirurgicale des tumeurs parotidiennes [25]. Les résultats de l´examen extemporané sont très variables et les série de cas sont souvent trop petites ou hétérogènes, ne séparant pas les tumeurs parotidiennes des autres tumeurs des glandes salivaires [26]. Sa sensibilité et sa spécificité rapportées dans la littérature varient respectivement de 40% à 100% et de 87% à 100% [26, 27] (Tableau 4), cette grande différence entre les études peut être expliquée par le fait que les résultats indéterminés ou atypiques sont considérés positifs ou négatifs. Wong [28] a considéré les résultats atypiques comme positifs, alors que Carvalho *et al*. [29] les a considérés comme négatif. Dans notre étude nous avons considéré les résultats indéterminés comme positifs, la sensibilité était 89% et la spécificité était de 88%. Le taux de faux positif rapporté dans littérature varie entre 0% et 12% [27]. Nos résultats sont comparables à ceux de la littérature. Le taux de faux négatif rapporté dans littérature varie entre 9% et 24% [28]. Nos résultats sont comparables à ceux de la littérature.

**Tableau 4:** sensibilité et spécificité de l’examen extemporané, rapportées dans la littérature comparée à notre série

Études	pays	Nombre de cas	Sensibilité	Spécificité
**Badoual et al 2006**	France	694	79%	99%
**Carvalho et al 1999**	Brésil	153	62%	98%
**Hwang et Brett 2003**	Singapour	36	100%	100%
**Ishida et al 1999**	Japon	152	93%	99%
**Iwai et al 1999**	Japon	167	96%	99%
**Longuet et al 2001**	France	94	75%	100%
**Arabi Mianroodi et al 2006**	Australie	30	100%	100%
**Seethala et al 2005**	Etats-Unis	61	74%	100%
**Tew et al 1997**	Australie	144	96%	99%
**Upton et al 2007**	Etats-Unis	155	96%	99%
**Wong 2002**	Hong Kong	19	100%	90%
**Zb?ren et al 2008**	Etats-Unis	110	93%	95%
**Zheng et al 1997**	Chine	65	88%	98%
**Notre série**	Maroc	72	89%	88%

## Conclusion

L´évaluation précise préopératoire des tumeurs de la glande parotide est devenue un véritable challenge pour les ORL et radiologues, essentiellement pour prédire la nature de la lésion et guider ainsi la décision thérapeutique. La distinction entre tumeur maligne et tumeur bénigne est parfois difficile surtout pour les tumeurs malignes de bas grade qui sont difficiles à différencier des tumeurs bénignes; mais aussi pour les adénomes pléomorphes de grandes tailles qui sont difficilement distingués des tumeurs malignes.

### Etat des connaissances sur le sujet

- La connaissance préopératoire de la nature de la tumeur guide le chirurgien dans sa décision chirurgicale; par conséquent, l´imagerie est devenue un véritable outil diagnostique pour les chirurgiens, l´échographie couplée ou non à la cytoponction, la TDM et l´IRM, sont les méthodes les plus fréquemment utilisées;
- Cependant, l´efficacité de chacune dans l´évaluation de la nature de la tumeur n´est pas encore bien définie.

### Contribution de notre étude à la connaissance

- Au terme de notre étude nous sommes arrivés à conclure à une sensibilité spécificité à l´IRM de 79% et 82% respectivement;
- Des faux négatifs et faux positifs de l´examen extemporané de 0-12% et 9-24%;
- D´où la nécessité de développer et généraliser les nouvelles techniques dynamiques de l´IRM telles que la mesure du coefficient de diffusion, et -MR spectroscopy- qui ont démontré des résultats prometteurs en matière de différenciation entre tumeur maligne et tumeur bénigne, et même dans le diagnostic étiologique: différencier entre adénome pléomorphe et tumeur de Warthin, et entre ces deux entités et les tumeurs malignes.
